# Exploring the added value of virtual reality biofeedback game DEEP in forensic psychiatric inpatient care—A qualitative study

**DOI:** 10.3389/fpsyg.2023.1201485

**Published:** 2023-11-09

**Authors:** Lisa Klein Haneveld, Hanneke Kip, Yvonne H. A. Bouman, Joanneke Weerdmeester, Hanneke Scholten, Saskia M. Kelders

**Affiliations:** ^1^Department of Research, Transfore, Deventer, Netherlands; ^2^Centre for eHealth and Wellbeing Research, Department of Psychology, Health and Technology, University of Twente, Enschede, Netherlands; ^3^HKU University of the Arts, Centre for Educational Research and Innovation, Utrecht, Netherlands; ^4^Games for Emotional and Mental Health (GEMH) Lab, Enschede, Netherlands; ^5^Communication Science, Department of Technology, Human and Institutional Behavior, University of Twente, Enschede, Netherlands; ^6^Opentia Research Unit, North-West University, Vanderbijlpark, South Africa

**Keywords:** DEEP, virtual reality, forensic mental healthcare, focus groups, interviews

## Abstract

**Background:**

Low motivation and suboptimal cognitive skills are common among forensic psychiatric patients. By focusing on doing and experiencing, innovative technologies could offer an alternative to existing treatment for this patient group. One promising technology is DEEP, a VR biofeedback game that teaches diaphragmatic breathing, which has shown its potential in reducing stress in other populations. This exploratory study aimed at identifying if, how and for whom DEEP can be of added value in forensic mental healthcare.

**Methods:**

This study used a qualitative approach. Six focus groups with 24 healthcare providers and 13 semi-structured interviews with forensic psychiatric inpatients were conducted in two Dutch forensic mental healthcare organizations. All healthcare providers and patients experienced DEEP before participating. The data were coded inductively, using the method of constant comparison.

**Results:**

The data revealed six themes with accompanying (sub)codes, including (1) the possible advantages and (2) disadvantages of DEEP, (3) patient characteristics that could make DEEP more or (4) less suitable and beneficial, (5) ways DEEP could be used in current treatment, and (6) conditions that need to be met to successfully implement DEEP in forensic mental healthcare. The results showed that DEEP can offer novel ways to support forensic psychiatric patients in coping with negative emotions by practicing diaphragmatic breathing. Its appealing design might be suitable to motivate a broad range of forensic psychiatric patient groups. However, DEEP cannot be personalized, which might decrease engagement and uptake of DEEP long-term. Regarding its place in current care, DEEP could be structurally integrated in existing treatment programs or used *ad hoc* when the need arises. Finally, this study showed that both healthcare providers and patients would need practical support and information to use DEEP.

**Conclusion:**

With its experience-based and gamified design, DEEP could be useful for forensic mental healthcare. It is recommended that patients and healthcare providers are included in the evaluation and implementation from the start. Besides, a multilevel approach should be used for formulating implementation strategies. If implemented well, DEEP can offer new ways to provide forensic psychiatric patients with coping strategies to better control their anger.

## 1. Introduction

In forensic mental healthcare, patients receive mandatory treatment due to their delinquent or criminal behavior, which could partly be explained by one or more psychiatric illnesses (Bloem et al., 2011; Meynen, 2017; Sygel and Wallinius, 2021). Treatment of this heterogeneous patient group can be complex, due to differences in type of offences and diagnoses, generally low treatment motivation, and often suboptimal cognitive skills, such as reading, writing and self-reflection (Drieschner and Boomsma, 2008; Svensson et al., 2015; Kip et al., 2018). All these factors result in a unique setting, with a need for treatment that is tailored to individual needs. While treatment of forensic psychiatric patients resulted in a reduction of recidivism rates, there is room for optimizing treatment outcomes and further reducing these rates (Delfin et al., 2019; Probst et al., 2020; Edberg et al., 2022). One of the commonly used treatment frameworks in forensic mental healthcare is Cognitive Behavioral Therapy (CBT). While CBT has been very effective in treating depression and anxiety in general mental healthcare, for treatment of aggression in (forensic) psychiatric patients it has been less effective (Del Vecchio and O’Leary, 2004; Saini, 2009; Henwood et al., 2015). Additionally, an earlier study concluded that forensic psychiatric inpatients do benefit from CBT regarding psychopathology and coping, but only a small group showed reliable change over time (Timmerman and Emmelkamp, 2005). A possible explanation for these results is that many CBT-based treatment methods rely on the ability of patients to think and talk about their behavior, which might be challenging for forensic psychiatric patients due to limited cognitive skills (Kip et al., 2019c; Kip and Bouman, 2020, 2021). These shortcomings of existing treatments highlight the need for novel strategies and interventions that better suit the needs, skills, and interests of forensic psychiatric patients.

eHealth – which is the use of technology to support health, wellbeing, and healthcare - can be used to improve care for forensic psychiatric patients (Kip et al., 2020a; Stoll et al., 2020; Tossaint-Schoenmakers et al., 2021). Over the last 10 years, an increasing number of eHealth interventions has been introduced in forensic mental healthcare (Kip et al., 2019b; Torous et al., 2020; Kirschstein et al., 2023). Many technologies that still rely on written text, such as web-based interventions and do not seem to fully fit the forensic psychiatric patient population (Kip et al., 2020b). However, experience-based technologies such as virtual reality, serious games and wearables with biofeedback can offer new, innovative possibilities to increase emotion regulation and wellbeing (Ilioudi et al., 2023; Kothgassner et al., 2023), and involve forensic patients and teach them the skills that are needed to prevent recidivism (Kip et al., 2019c; Kip and Bouman, 2020, 2021). A Dutch study with 110 forensic psychiatric healthcare providers and patients demonstrated the enthusiasm for these technologies, particularly because of their immersive qualities and focus on “doing and real-time experiencing,” instead of “thinking and talking” about behavior (Kip et al., 2019c). These findings indicate that an experience-based, gamified approach accounts for the low literacy- and treatment motivation levels of the forensic population. Despite the apparent potential, not much research has been conducted on the added value of these experience-based, gamified technologies in forensic mental healthcare.

**FIGURE 1:**
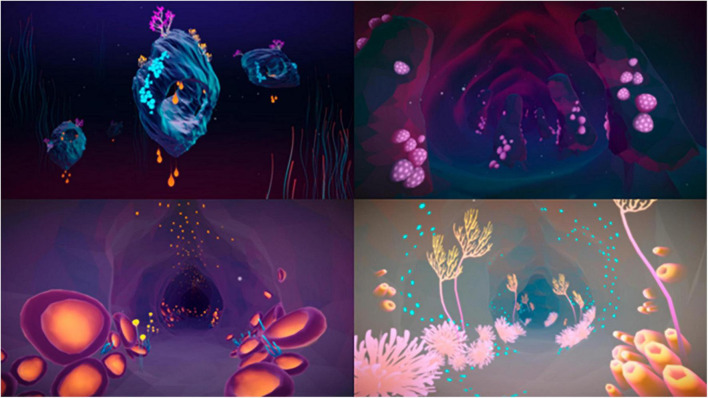
Visual examples of DEEP. Reproduced with permission from Development Team Explore DEEP (https://exploredeep.com).

Over the years, virtual reality (VR) has become a growing topic of interest within psychological therapy (Rizzo et al., 2018; Sygel and Wallinius, 2021; Kothgassner et al., 2023; Kouijzer et al., 2023). In VR, it is possible for the user to be physically present and interact with a virtual environment in an engaging way (Kim et al., 2017; Pillai and Mathew, 2019). Additionally, while more research on the effectiveness is necessary, VR seems to be a potentially suitable tool for treatment of aggression (Kip et al., 2019b; Klein Tuente et al., 2020; Smeijers et al., 2021). An example of such a VR-application is DEEP. This experience-based and gamified VR technology provides a unique combination of breathing techniques and biofeedback to teach its player diaphragmatic (or deep) breathing in an intuitive and engaging manner to reduce stress and anxiety (Weerdmeester, 2021). By applying deep breathing, the user “swims” through a fictional virtual underwater environment and explores colorful caves while following a, by artists designed, route (see Figure 1). The biofeedback in DEEP can provide users with real-time information about their breathing by using a waistband that measures the movement of their diaphragm and by showing them visualizations of how well they are inhaling and exhaling (e.g., corals that light in DEEP and visual breathing circles). From a practical point of view, DEEP takes the relatively little time and technical skills of healthcare providers (Weerdmeester, 2021). This might make DEEP it easier to adopt than for instance interactive VR with roleplaying functionalities. Additionally, DEEP is designed to be an appealing and gamified environment, which might positively affect treatment motivation (Kip et al., 2019b). Moreover, DEEP is designed to be a calm environment for users to relax and decrease stress, which could be a helpful way for psychiatric patients to increase their wellbeing when a physical calm room is not available (Ilioudi et al., 2023). Hence, DEEP might provide possibilities to add something entirely new to forensic treatment, because of its emphasis on deep breathing and providing real-time biofeedback to its user. This is often not possible in standardized treatment, where the patient must rely on their memory and ability to reflect on past experiences and behavior. While DEEP has not yet been studied in the forensic psychiatric population, it seems that an interventions like DEEP can be of added value for the forensic mental healthcare due to its use of biofeedback, deep breathing, and its immersive, gamified design.

Due to its working mechanisms, it DEEP might be a useful approach to prevent aggression. For example, studies have shown that biofeedback can be helpful in reducing aggression. To illustrate, interventions based on heart rate (variability) biofeedback can decrease anger in adolescents and improve emotion-regulation in offenders, which is an often-re-occurring treatment goal for forensic psychiatric patients (Savard, 2017; Gray et al., 2019). Moreover, diaphragmatic breathing has shown to be supportive in reducing aggressive behavior in various psychiatric patient groups, by modulating the heart rate and specific neural circuits that are involved in emotion regulation (Gillespie et al., 2012; Phillips et al., 2019). Regarding the effectiveness of DEEP: recent studies have also shown DEEP to be effective in reducing stress and anxiety in students and in showing less disruptive behavior in adolescents with behavioral problems (Van Rooij et al., 2016; Bossenbroek et al., 2020). Despite the promising results of biofeedback in general and DEEP in specific settings, it is unclear if and how DEEP can be used by forensic psychiatric patients and support them in their treatment outcomes (e.g., reduce stress and anger, or increase emotion-regulation). Likewise, the implementation of VR in forensic mental healthcare can be challenging, due to the necessity of healthcare providers to develop the right attitude and skill set to integrate VR in their treatment (Kip et al., 2020a; Tossaint-Schoenmakers et al., 2021; Kouijzer et al., 2023). Therefore, it is important to gain insight into how to successfully implement DEEP in forensic mental healthcare.

### 1.1. The current study

The objective of this study is to identify if and how DEEP can be of added value for forensic psychiatric inpatient settings according to healthcare providers and patients in forensic psychiatric inpatient care. The four accompanying research questions are to explore (1) expected advantages and disadvantages of DEEP in forensic mental healthcare, (2) for which types of forensic psychiatric inpatients DEEP could be most effective, (3) in what ways DEEP could be used in current treatment of inpatients, and (4) which factors are important for implementing DEEP within clinical forensic mental healthcare, according to healthcare providers and patients.

## 2. Materials and methods

### 2.1. Study design and setting

In the current study, focus groups with healthcare providers and semi-structured interviews with patients were conducted to answer the research questions. The Consolidated criteria for Reporting Qualitative research (COREQ) guidelines were used to report the focus groups and interviews (Tong et al., 2007). Before conducting the focus groups and interviews, participants verbally received information about DEEP as well as the scope of the study. Additionally, it was ensured that the forensic psychiatric patients understood that participating on this study was voluntarily and that it was not part of their treatment program. With support from the researcher (LK), who was already trained in using DEEP, all participants were able to test DEEP to experience the underlying mechanisms beforehand. This was ethically feasible, because during earlier studies in which participants used DEEP no severe physical risks were found, except for some motion sickness that can occur when someone is not used to VR (Bossenbroek et al., 2020; Weerdmeester et al., 2021). Ethical approval was given by the Ethics Committee of the University of Twente (Behavioral, Management and Social Sciences, nr. 21119).

This study has been conducted at inpatient clinics of two forensic mental healthcare organizations: Transfore and De Woenselse Poort. Both organizations offer forensic mental healthcare to in- and outpatients who have committed or are on the verge of committing a criminal offence, due to their psychiatric problems. Transfore has multiple locations in the east of The Netherlands and offers treatment to over 1,500 patients per year. De Woenselse Poort is based in the south of The Netherlands and provides treatment for around 550 patients per year. In total, six inpatient clinics (three per organization) participated.

### 2.2. DEEP

**FIGURE 2:**
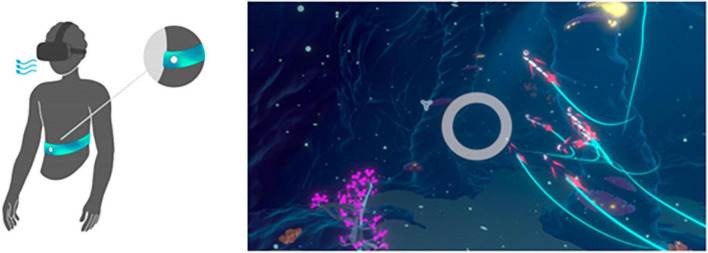
Waistbelt and wide circle to provide biofeedback to DEEP user. Reproduced with permission from Development Team Explore DEEP (https://exploredeep.com).

DEEP is a VR-game that uses biofeedback to teach relaxation skills to its user and is based on scientific knowledge about anxiety and stress regulation (Van Rooij et al., 2016; Weerdmeester, 2021). The player is placed in a surrealistic and immersive underwater world that they can move through by using their own diaphragmatic -or deep- breathing. During DEEP, the player wears a waistband that continuously measures their breathing. Within the game, a wide circle in front of the player mirrors the breathing of the player (see Figure 2). Additionally, the corals and plants in the world grow, shrink, and change in illumination with every inhale and exhale. Both techniques provide feedback on changes in physical signals, in this case, the way someone is breathing, which is called biofeedback (Weerdmeester, 2021). By deep breathing via their diaphragm, the player can move through the game, while shallow upper-chest breathing -which often happens in stressful situations- is not or barely measured by the waistband. Therefore, shallow breathing hinders the progress in the game, while diaphragmatic breathing results in progress. As a result, the player has continuous insight in their own progress and whether their breathing needs adjustment. By learning deep breathing in a gamified and engaging way, the user might become able to better cope with negative emotions and increase their wellbeing (Tellhed et al., 2019; Ahmadpour et al., 2020; Weerdmeester et al., 2021). In this study, the same version of DEEP was used as the one used in an earlier conducted randomized controlled trial (Weerdmeester et al., 2021). The version was built for the *Oculus Rift dk1* headset and *HTC vive*.

### 2.3. Participants

In this study both healthcare providers and patients were included since they will have an active role in the use of DEEP. Healthcare providers will have a role in introducing DEEP and supporting patients, while patients are the end-users of DEEP. All healthcare providers directly involved in any treatment, support or supervision of inpatients were eligible to participate and were included via convenience sampling. With help of managers and the use of information folders, the study was introduced to the inpatient healthcare providers. Patients were recruited via the healthcare providers who participated in the focus groups. They were asked to inform their patients about the study and distribute information flyers. Patients were excluded from participation if they were diagnosed with a current psychosis or if a therapist indicated that they were not able to participate for any relevant reason.

### 2.4. Materials and procedure

Six focus groups with healthcare providers and thirteen interviews with patients were conducted between November 2021 and April 2022 by one researcher (LK) and took place at the location of the forensic mental healthcare organization where the healthcare providers were working, and the patients received treatment.

#### 2.4.1. Focus groups

Each focus group with healthcare providers took around 60 min (min = 43, max = 71). At the start of the focus groups, participants were informed about the design and mechanisms of DEEP as well as the scope of this research. After the introduction of the goal and structure of the focus group, all participants signed the informed consent. In the first part of the focus groups each participant was able to use DEEP for a couple of minutes to gain an impression of if and how the biofeedback in DEEP changed the way they were breathing. During this part, all caregivers tried out DEEP. However, one caregiver only used DEEP for a minute as they experienced motion sickness.

In the second part of the semi-structured focus group, a topic list was used to explore the first impressions of DEEP (see Appendix A) and elicit scenarios of when, how and for whom DEEP could be used in forensic treatment. This topic list was developed by the researchers and evaluated by the other project members. During the focus groups, the participants were able to express their first impression of DEEP and what they liked and disliked about using it, using questions as *“To what extent do you think that DEEP can be of added value for forensic treatment?”* They were also asked about the advantages and added value DEEP might have for the forensic psychiatric treatment, and specifically for what type of patients. An example of a question is: *“What type of patients would benefit most from using DEEP?”* Finally, the participants answered questions about possible barriers of using DEEP as well what they found important preconditions for implementing DEEP. For example: *“When implementing DEEP in your forensic clinic, what factors are important to take into account?”*

#### 2.4.2. Interviews

The semi-structured interviews with patients took around 20 min (min = 15 and max = 27 min). The interviews used the same themes from the topic list that was used in the focus groups, but were kept short and concise, which fits the skills and attention span of most forensic psychiatric patients (Kip et al., 2019a,^2022^). Before conducting the interview, the participating patients were given a brief introduction about DEEP and the interview. During this introduction, the researcher verbally explained the content provided in the information folder in short sentences, allowing patients of all cognitive levels to understand the scope of the study and interview. All participants were also given the opportunity to ask questions. After this introduction, the participants signed the informed consent, which consisted of short statements for them to read. After the informed consent was signed, the patients were able to become acquainted with DEEP for several minutes. They did so in the same way as the caregivers. Only one patient had trouble using DEEP and stated to feel scared. However, after a moment of standing still and getting used to the environment, they were able to move around and experience DEEP.

In the second part of the interview, a topic list was used (see Appendix B), which was evaluated beforehand by caregivers specialized in working with forensic psychiatric patients with lower cognitive abilities. The semi-structured approach allowed the researcher to ask probing questions, deviate from the order of questions, or tailor them depending on the patient’s cognitive skills and attention span. Patients were asked questions about their first impression of DEEP and which advantages they thought it might have for them via questions such as: *“Which advantages do you think DEEP might have for you? Could it help you?.”* They were also asked if they were willing to use DEEP more often and if so, when. An example question is: *“On which moments or periods would you like to use DEEP?”* Moreover, questions were asked about possible disadvantages and when patients are not willing to use DEEP. Finally, they were asked about considerations before implementing DEEP and what they would require before they would be able to use DEEP.

### 2.5. Data analysis

All focus groups and interviews were audio-recorded, transcribed verbatim and coded inductively. By using the method of constant comparison (Boeije, 2002), the raw data from the focus groups and the interviews were organized into categories after which codes were created to connect the fragments in each category. The coders (LK and HK) used Microsoft Excel to code the fragments as it is a simple and cost-effective way to thematically and inductively code qualitative data (Bree and Gallagher, 2016). First, one coder (LK) read the transcripts to become familiar with their content. Second, all fragments related to one of the research questions were selected and divided over each research question: (dis-)advantages of using DEEP (RQ 1), characteristics of patients that would or would not benefit of using DEEP (RQ 2), ways of using DEEP in treating patients (RQ 3) and conditions of implementing DEEP in forensic mental healthcare organizations (RQ 4). Based on these fragments, one coding scheme for every research question (theme) was created inductively: the main and subcodes, with accompanying definitions, were formulated using a bottom-up approach, using the content of the fragments. Several main codes and subcodes were identified and used to code the fragments. Throughout this process, the coding schemes and definitions were constantly adapted and updated by two researchers (LK and HK), using an iterative approach. After coding four focus groups and nine interviews, data saturation was reached and no new codes were identified. The first coder used the coding schemes to code all fragments, which then was sent to the second coder (HK) to code 20% of the fragments independently with an agreement rate of 78%, which is acceptable in the coding process (Miles and Huberman, 1994). The disagreement between the two coders was discussed until consensus was reached, and minor adaptations were made to the coding schemes accordingly.

## 3. Results

### 3.1. Demographics

For this study, 24 healthcare providers and 13 patients were included. Regarding the healthcare providers, 22 were included in 6 focus groups with an average of four participants per group (min = 2, max = 6). Four focus groups took place at Transfore (*n* = 14) and two at De Woenselse Poort (*n* = 10). Two of the 24 healthcare providers were interviewed individually, because they were not able to join the organized focus groups but were still interested in using DEEP and sharing their experiences. Of all participating healthcare providers, 7 identified as male and 14 as female. All healthcare providers worked at an inpatient clinic: one healthcare provider was a psychologist, one a coordinating therapist, two were team managers, two were forensic nurses in training and 18 healthcare providers were socio-therapists/forensic nurses. All interviewed patients received inpatient care, of which 11 identified as male and 2 as female. An overview of the included healthcare providers and patients per forensic inpatient clinic are provided in Appendix C.

### 3.2. Possible advantages of using DEEP

Healthcare providers and patients were asked to provide their view on DEEP and the possible advantages it might have for forensic inpatients. The participants provided possible advantages on the content as well as the design of DEEP. The identified codes are provided in Table 1.

**TABLE 1:** Possible advantages of using VR-DEEP, according to healthcare providers (*n* = 24) and patients (*n* = 13).

Codes	Definition of code	Codes*^a^*	Healthcare providers*^b^*	Patients*^c^*
**Advantages–content**				
Relaxation and emotion regulation	Playing DEEP contributes to a feeling of relaxation and rest and helps the player in recognizing and dealing with arousal, stress or anger	34	4 (8)	10 (26)
Deep breathing	Playing DEEP contributes to the recognition and practice of diaphragmatic breathing	23	13 (16)	4 (7)
Meditation and Mindfulness	The principles of DEEP are reminding of principles of meditation and Mindfulness, e.g., the focus on the body and breathing combined with the surrounding and music	6	2 (2)	1 (4)
**Advantages–design**				
Appealing design	In DEEP the player has a fun and extraordinary experience by stepping into a surrealistic and relaxing environment	56	18 (25)	9 (31)
Accessible	DEEP requires little cognitive and reflective skills and can be used by everyone	14	11 (13)	1 (1)
User-friendly	DEEP can be set up easily, quickly and independently	3	2 (3)	

^a^The total number of times a code was mentioned in all focus groups and interviews. ^b^The number of different healthcare providers that mentioned a code, and (#) the total number of times the code was found in all focus groups. ^c^The number of patients that mentioned a code, and (#) the number of times the code was found in all interviews with patients.

Regarding the content of DEEP, both healthcare providers and patients indicated that playing DEEP might be able to contribute to more *relaxation and better emotion regulation*. Four healthcare providers and 10 patients thought that DEEP might predominantly help to relax and de-stress during tense situations or after conflict. Second, nine patients mentioned that they often feel stress, while seven of them added that using DEEP made them feel more relaxed.

Second, DEEP might help focus on *deep breathing.* During the focus groups, 13 healthcare providers acknowledged DEEP to be a useful intervention in learning patients to breathe slowly and deeply, which might help them to de-stress and cope with stressful or emotional situations in the future. Additionally, four patients stated that focusing on their breathing was a new but useful experience to find relaxation.

A third code that was identified by two healthcare providers is that DEEP might be able to introduce the player to principles of other types of interventions such as *mindfulness and meditation*, in a down-to-earth and playful manner, which might make it easier for patients to open themselves up to it. This view was acknowledged by one patient in stating the following: *“There are men here that like mindfulness and meditation and use it in their daily lives. But there are also men who find it rubbish. Maybe DEEP can convince them that getting some rest in your mind is really good and does not have to be boring.”*

As for the design features of DEEP, 18 participants of the focus groups found DEEP to have an *appealing design*, which might help increase treatment motivation of forensic inpatients. Ten healthcare providers illustrated that this group often shows low treatment motivation and are not always willing to try new interventions. Because DEEP is developed as a game and is meant to provide a relaxing experience, patients might become more enthusiastic to try new interventions that are focused on experiencing, instead of reflecting. Participant 24 indicated the following regarding the suitability of the design of DEEP for forensic inpatients:” DEEP *does not ask you to do anything, except doing something you always do, namely breathing, and at the same time you learn to focus on all kinds of internal things unconsciously. While doing something fun. Yes, I do think that is especially fitting for our patient group.”* According to nine patients, DEEP is a new, fun, and relaxing experience that they do not always get to have in their current treatment. Some added that DEEP enabled them to step into another world, where they can swim, steer, and breathe, which felt like an enjoyable experience.

Second, 11 healthcare providers mentioned that DEEP is *accessible* for everyone and does not require much cognitive skill, which is a great advantage for the forensic patient group. Participant 5 stated the following regarding the accessibility of DEEP: *“I think that it is a game with many features. But it functions also as a resting place, which asks relatively little of you as a player.”* Finally, two healthcare providers appreciated that DEEP is *user-friendly* and that it can be started up easily and quickly, which enhances the chance that healthcare providers will structurally use an intervention with their patients.

### 3.3. Possible disadvantages of using DEEP

Additionally, to the first category, healthcare providers and patients were asked to provide their insight on possible disadvantages of DEEP for forensic inpatients. The identified codes are provided in Table 2.

**TABLE 2:** Possible disadvantages of using VR-DEEP, according to healthcare providers (*n* = 24) and patients (*n* = 13).

Codes	Definition of code	Codes^a^	Healthcare providers^b^	Patients^c^
**Disadvantages of DEEP**				
Simple design	DEEP has a simple and straight-forward design, which might affect immersion negatively and becomes boring	(24)	5 (8)	8 (16)
Suboptimal controls and biofeedback	The controls and biofeedback of DEEP is dated and does not always work properly, e.g., the waistbelt and the in-game visuals	(18)	8 (10)	5 (8)
Overwhelming design	DEEP requires the player to look around, steer, breath and process visual feedback, which might overwhelm or induce stress	(7)	3 (3)	2 (4)
No personalization	It is not possible to choose other worlds or levels in DEEP and there are no available triggers that can be personalized	(4)	2 (3)	1 (1)
Many wires	DEEP needs many wires to connect the VR-set, waistband and sensor to a laptop, obstructing the player to rotate properly	(3)	3 (3)	
VR motion sickness	DEEP can induce motion sickness or dizziness to its player	(2)	2 (2)	
No clear connection with daily life	In DEEP diaphragmatic breathing is trained in a surrealistic environment, with no direct connection to real life situations.	(2)	2 (2)	
Expensive	Purchasing DEEP and a VR-set is expensive	(1)	1 (1)	

^a^The total number of times a code was mentioned in all focus groups and interviews. ^b^The number of different healthcare providers that mentioned a code, and (#) the total number of times the code was found in all focus groups. ^c^The number of patients that mentioned a code, and (#) the number of times the code was found in all interviews with patients.

As a first disadvantage of DEEP, five healthcare providers mentioned that DEEP might not be able to engage the player long term, because of its *simple and straight-forward design*. During the focus groups, it was mentioned that the new generation inpatients is really into new gaming technology and might find DEEP too simple or too easy to keep emerging themselves after they used DEEP a couple of times. Eight patients shared this view and one of them illustrated it as following: *“In DEEP, you can only just swim around, without being able to communicate with the world around you. You cannot do anything or touch anything. I feel like that might become boring after a while.”*

Second, eight healthcare providers indicated that the *controls and biofeedback felt suboptimal* or dated. Additionally, five patients mentioned that the waist belt did not always give them correct feedback on their breathing. This was again confirmed by some healthcare providers, of whom some also stated that the biofeedback that was given in-game (e.g., corals that light up with every breath), was sometimes hard to perceive and connect to what the player was doing. This is illustrated by participant 3: *“I am wondering if DEEP should give more visual stimuli, so that questions like “am I doing alright?” are answered. During DEEP I was able to focus on my breathing, but for our patient group it is good to clearly experience what happens if they are breathing “well” or “bad” If it stays subtle it might become too abstract for a part of our patient group.”*

Third, three healthcare providers indicated that DEEP could be *overwhelming* for some of their patients, because it focuses on many features: breathing, steering, and looking around while processing the biofeedback. A couple of patients stated that they were overwhelmed, especially those who never experienced virtual reality before.

Fourth, two healthcare providers mentioned the *lack of personalizatio*n in DEEP, with no available triggers for different patients. They stated that for some patients it might be helpful to use more triggers in DEEP, so that they can train their deep breathing during a tense situation. For other patients it might help to have a less dark and surreal environment because they are easily overwhelmed.

In this study, an older version of DEEP was used that needed wires to connect a laptop to the VR-set and the waist belt. Hence, three healthcare providers felt that the *many wires* might obstruct someone to move properly while playing DEEP. Two healthcare providers were also wondering if playing DEEP could not induce *motion sickness*. Two other healthcare providers were questioning if the design and mechanics of DEEP *lack a clear connection to daily life*. Because DEEP uses a surreal environment it might become difficult for patients to apply the things they learn in their real-life situations. Finally, one healthcare provider mentioned that purchasing DEEP might be too *expensive*, when compared to other interventions, such as free apps that focus on meditation and deep breathing.

### 3.4. Patients that might benefit from using DEEP

The third main category of codes relates to patient groups that might benefit most from using DEEP. Only a couple of patients were able to indicate which patient groups might or might not benefit from DEEP. Most patients stated that they had a hard time predicting which patients would like DEEP or might find DEEP useful. They reported that they just focused on themselves and did not feel like interfering in other patients’ treatment. The identified codes are provided in Table 3.

**TABLE 3:** Possible patient characteristics that might benefit from DEEP, according to healthcare providers (*n* = 24) and patients (*n* = 13).

Codes	Definition of code	Codes^a^	Healthcare providers^b^	Patients^c^
**Patient characteristics that might benefit**				
Everyone who has an open mind	Not only forensic patients, but everyone can benefit from DEEP, as long as one is open to the effects of breathing and relaxation exercises.	10	7 (9)	1 (1)
Emotion regulation issues	Patients who have difficulty controlling their emotions	8	7 (8)	
Body signals recognition issues	Patients who have difficulty recognizing and verbalizing their body signals	5	4 (5)	
Suboptimal cognitive skills	Patients with less optimal cognitive skills	4	4 (4)	
Personality issues	Patients with personality issues, such as borderline personality disorder or antisocial disorder	3	2 (3)	
Introversion	Patients who are introverted or are not comfortable talking about what they are experiencing	2	1 (1)	1 (1)

^a^The total number of times a code was mentioned in all focus groups and interviews. ^b^The number of different healthcare providers that mentioned a code, and (#) the total number of times the code was found in all focus groups. ^c^The number of patients that mentioned a code, and (#) the number of times the code was found in all interviews with patients.

First, nine healthcare providers indicated that almost everyone might benefit from using DEEP, patient, or non-patient. However, seven of them added that it is essential that the *user has an open mind* toward the idea of deep breathing and using it as a coping mechanism in reducing negative emotions.

Second, seven healthcare providers mentioned that patients who have *difficulties in regulating their emotions* could benefit from DEEP, especially those with stress, anxiety, and reactive aggression. This is further explained by the following quote of participant 7: *“I think that patients who feel tense really quickly and therefore can react impulsively might benefit from DEEP. For example, (name patient) who tried DEEP, is quite impulsive in his behavior and reactions. And I saw how DEEP affected him and how he felt more relaxed. So, for patients like him DEEP could be a good fit.”*

Third, four healthcare providers indicated that DEEP could help patients who have *issues with recognizing their bodily signals* in helping them to focus on their breathing and what they are experiencing while doing that. Participant 21 illustrated this as following: *“It seems to me that this is a good game for most of our patients. They sometimes have trouble pin-pointing their feelings. By using DEEP they can just experience their feelings, while focusing on their breathing.”*

Fourth, four healthcare providers mentioned that patients with *suboptimal cognitive skills* could benefit from using DEEP. They stated that DEEP is easy to use, and its goal can be understood without relying on reading, writing, or reflecting skills of its user.

Fifth, two healthcare providers indicated that DEEP can be useful for people with issues *regarding their personality*, because they are not able to manipulate the situation, but must put trust in the intervention and their own breathing. Finally, one healthcare provider and one patient mentioned patients who are *introverted* as a group that might benefit from DEEP. This patient stated the following: *“In DEEP you can just be in your own world. You are able to artificially open yourself to the experience, without having to share it with others if you don’t want to. That might provide a shelter for someone to go back to and de-stress.*”

### 3.5. Patients that might benefit less from using DEEP

The fourth category relates to patient groups that might benefit less from using DEEP. An overview of this category and the identified codes are given in Table 4.

**TABLE 4:** Possible patient characteristics that might less benefit from DEEP, according to healthcare providers (*n* = 24) and patients (*n* = 13).

Codes	Definition of code	Codes^a^	Healthcare providers^b^	Patients^c^
**Patient characteristics that might benefit less**				
Psychotic vulnerability	Patients who have psychotic sensitivity or are having a psychotic episode	7	5 (7)	
Epileptic sensitivity	Patients who are sensitive for epileptic episodes	3	1 (2)	1 (1)
(Game)addiction sensitivity	Patients who have (game)addiction sensitivity or who tend to obsessively use new things	2	1 (1)	1 (1)
Balance issues	Patients who have issues in their balance, which makes them sensitive for dizziness or motion sickness	1	1 (1)	

^a^The total number of times a code was mentioned in all focus groups and interviews. ^b^The number of different healthcare providers that mentioned a code, and (#) the total number of times the code was found in all focus groups. ^c^The number of patients that mentioned a code, and (#) the number of times the code was found in all interviews with patients.

Sixteen healthcare providers provided no specific patient group that would not benefit from DEEP. Five healthcare providers were wondering whether patients with *psychotic vulnerability* could benefit from DEEP. They indicated that those patients might become overwhelmed or paranoid, which is illustrated by the following quote: “*I might not use DEEP with patients who are in a psychotic, paranoid state. If someone is totally shielded by the VR-headset he or she has to trust their surroundings. They might wonder what would happen if they surrendered to another reality, which is necessary while playing a VR-game.”*

Additionally, one healthcare provider mentioned that patients with *epileptic sensitivity* might not benefit from DEEP, because of the flashing lights of the corals and plants in the game. Another healthcare provider mentioned *(game) addiction sensitivity* as something to keep in mind as some patients who are suffering from this seem to be more interested in finding new ways to distract themselves, than using the intervention to learn something new. One patient acknowledged this as well and stated the following: *“Of course it would be nice if you can just step into a room and put DEEP on your head. But with these kinds of people, you have to be careful that it does not become something like drugs, that it occupies their mind 24/7.”*

Finally, one healthcare provider indicated that patients with *balance issues* might not benefit from DEEP or virtual reality in general, as they might become disoriented.

### 3.6. Possible ways DEEP could be used in current forensic care

Healthcare providers and patients were asked about possible ways DEEP could effectively be integrated in current forensic care. The identified main and subcodes are provided in Table 5.

**TABLE 5:** Possible ways DEEP could be used in current treatment, according to healthcare providers (*n* = 24) and patients (*n* = 13).

Codes	Definition of code	Codes^a^	Healthcare providers^b^	Patients^c^
**Use of DEEP in practice**				
*Ad hoc* on patient initiative	Using DEEP as a flexible tool that provides patients the chance to use DEEP on their own initiative when they feel they need it.	19	9 (10)	7 (9)
Complementing existing breathing exercises	Using DEEP as a complementary tool to make current breathing exercises more compelling and accessible.	12	6 (9)	2 (3)
Structural within treatment	Using DEEP as a structural tool at a fixed moment (in treatment), where patients can train their deep breathing and emotion regulation, without feeling tense.	11	5 (7)	3 (4)

^a^The total number of times a code was mentioned in all focus groups and interviews. ^b^The number of different healthcare providers that mentioned a code, and (#) the total number of times the code was found in all focus groups. ^c^The number of patients that mentioned a code, and (#) the number of times the code was found in all interviews with patients.

All healthcare providers and most patients mentioned one way how DEEP could be used in current forensic care. First, nine healthcare providers and seven patients mentioned that DEEP could be introduced *ad hoc* by a healthcare provider or used on patients’ own initiative. This way DEEP can be used as a flexible intervention that can provide direct support to patients when they experience anger, stress, or anxiety. When using this approach, it might be possible to decrease these types of negative emotions in a short period of time. A patient illustrates this in the following quote: *“I would like to use DEEP when I need it, for example that I make a reservation for DEEP for 1 h. Because if I use DEEP when I do not need it and can’t use DEEP when I do, I might feel like crap the rest of the week.”*

Second, six healthcare providers and two patients indicated that DEEP can *complement already existing breathing exercises* that are provided in current treatment. More clinics use breathing exercises in their treatment plan, however many patients are hesitant to participate, because they find it too abstract or boring. According to the healthcare providers, DEEP could help to introduce deep breathing to patients in an engaging and accessible way.

Finally, five healthcare providers mentioned that DEEP could be used structurally, which meant multiple times per week on fixed moments. They stated that when using DEEP structurally, the patient can train their deep breathing without feeling tense or emotional, while learning to use their deep breathing as a coping skill when stressful situations do occur. The following quote of participant 6 illustrates this: *“It would be nice to use DEEP structurally. Then you are able to start with arousal and stress regulation. And it is something to put in the treatment plan. It means that someone who might feel OK at the moment is able to practice with DEEP. When someone feels that their tension is high, you are able to rely on the things and skills you have built upon together.”* Three patients agreed with the structural use of DEEP. They stated that using DEEP on fixed moments during the week or within therapy might give them a sense of support and structure.

### 3.7. Conditions for implementing DEEP within forensic mental healthcare

Finally, healthcare providers and patients were asked to provide possible conditions that should be considered when implementing DEEP within forensic mental healthcare. The identified codes are provided in Table 6.

**TABLE 6:** Possible conditions for implementing DEEP in forensic mental healthcare, according to healthcare providers (*n* = 24) and patients (*n* = 13).

Codes	Definition of code	Codes^a^	Healthcare providers^b^	Patients^c^
**Implementing DEEP**				
Support for patients	Healthcare providers must be able to support and instruct their patients, both before, during and after using DEEP, so patients can gain the confidence to discover DEEP	17	11 (12)	5 (5)
Room to play DEEP with no distractions	An available room must be provided that has a closing door and curtains, so patients can use DEEP undisturbed	16	9 (12)	4 (4)
Training for healthcare providers	A proper DEEP-training must be provided to all healthcare providers, so that they have the skillset to use DEEP with their patients (in their current treatment)	6	5 (6)	
Clear agreements on the usage of DEEP	Before implementing DEEP, healthcare providers and management need to make clear agreements on how, when and for whom DEEP will be used	5	4 (4)	1 (1)
Locks to prevent theft or vandalism	The VR-area should be secured in preventing theft or vandalism of the VR-set(s)	5	2 (2)	3 (3)
Good (spinning) desk chairs	To use DEEP optimally a good desk chair must be provided that has wheels and can rotate	4	3 (3)	1 (1)
Time to discover DEEP together	Healthcare providers need time and room in their agenda to discover together how DEEP can be used most effectively	1	1 (1)	
User support for healthcare providers	Healthcare providers need a helpdesk or support line where they can get information or help when using DEEP	1	1 (1)	

^a^The total number of times a code was mentioned in all focus groups and interviews. ^b^The number of different healthcare providers that mentioned a code, and (#) the total number of times the code was found in all focus groups. ^c^The number of patients that mentioned a code, and (#) the number of times the code was found in all interviews with patients.

First, 11 healthcare providers mentioned that it is important that *patients receive support* and instructions from their healthcare providers, especially when they use DEEP for the first time. Many healthcare providers stated that it is important to increase the confidence of patients while using DEEP, so that they feel it can help them in their treatment goals. Five patients indicated that DEEP can feel overwhelming: receiving some instructions before and support during DEEP might help them to stay calm. This is illustrated in the following quote by a patient: *“I would like some instructions and support. I feel like I might enjoy it even more, (*…*). But sometimes it is good to try something out and fail, this is how life works as well. But with some instructions I might have enjoyed it more. I also think I might have had a different kind of breathing throughout the whole game.”*

Second, nine healthcare providers and four patients indicated the importance of using DEEP in a quiet *room with no distractions*. Some healthcare providers mentioned that it is important to use DEEP in private, because many patients are easily distracted or feel uncomfortable by their peers. Participant 2 provided the following quote illustrating this topic: *“Well, I think that you should take privacy into account. Because when you use a VR-headset you are kind of in your own world. Many boys in our group were bullied and are hesitant to really emerge themselves, so we must provide room for them to do so.”*

Third, five healthcare providers mentioned the need for *training for all healthcare providers*, so that everyone in the clinic has the confidence and the skills to use DEEP with their patients. Fourth, four healthcare providers indicated that before implementing DEEP, management and the healthcare providers should make *clear agreements* to ensure that everyone knows why and for which type of patient groups DEEP is implemented, and in what way it can be used in inpatient care. These agreements should be visible for all colleagues to ensure that no clinic is hesitant to start using DEEP. One patient gave the following statement regarding this topic: *“All these interventions are good but should also be concrete. Sometimes you can talk and talk and talk, and still, it takes forever before something is available and useful. And if something is available, it is put away, because someone has to stay with the patient, and no one has time for it. These things should be taken into account, because there are many great projects, but the whole system you put it in, is really bureaucratic.”*

Fifth, two healthcare providers and three patients stated that it is necessary to make sure the *VR-area with DEEP can be secured safely*. Some of them shared their experience with new equipment that got stolen or vandalized. Sixth, three healthcare providers and one patient mentioned the need for a *good desk chair*, so that the user can move through DEEP without straining their neck. Seventh, one healthcare provider indicated the importance of providing *time in their agenda* to introduce DEEP to their patients and discover the intervention at their own pace. Finally, one healthcare provider shared the need for *user support* for when they need information or have questions regarding DEEP.

## 4. Discussion

### 4.1. Answering the research questions

The aim of this study was to gain insight in the possible added value of DEEP for treatment of forensic psychiatric inpatients. First, several possible advantages of using DEEP in forensic psychiatric inpatient clinics were identified. First, participants especially valued the promise DEEP has for engaging unmotivated patients, by using a gamified approach and an appealing design. Moreover, by its focus on the experience in a VR-environment with no use of written text or assignments, participants expected DEEP to resonate with patients with suboptimal cognitive skills. However, the results also showed some points of improvement of DEEP. Participants indicated that the hardware of DEEP felt in some ways suboptimal: the VR-controls and -headset showed some malfunction and multiple wire connections were necessary before using DEEP. Additionally, some design features of DEEP might not fit the skills of forensic patients, according to the participants. They found that DEEP uses a straight-forward design, but still requires multitasking from its user while playing. Participants wondered if this could lead to frustration in some forensic inpatients. Second, most healthcare providers concluded that DEEP might be useful for any type of patient if they are willing to have an open mind to the effects of diaphragmatic breathing on their wellbeing. Some patients’ characteristics, like psychotic vulnerability, were mentioned as potentially unsuitable for using DEEP by several participants, due to its surrealistic and immersive design. Patients were less able to provide insight into specific patient groups that might benefit from using DEEP because they were mostly preoccupied with their own treatment. Third, participants shared several views about how DEEP could be integrated into current forensic practice. On the one hand, DEEP could be used on structurally (e.g., on fixed days) aimed at the acquisition of skills, such as using deep breathing to cope with negative emotions. On the other hand, DEEP could be used *ad hoc* as a short-term relief of anxiety or anger or as an intervention for when patients are preparing for a challenging task during their treatment. Finally, many practical preconditions were given for implementing DEEP in forensic mental healthcare. For the implementation of DEEP to be successful, our study emphasized the needs of patients and healthcare providers, such as practical support, sharing of information, clear and concise instructions, and scheduled time to include DEEP in their daily practice.

### 4.2. The added value of DEEP in forensic mental healthcare

One of the main findings of this study is that healthcare providers and patients stated that DEEP has the potential to add something new and unique to the forensic mental healthcare. In an earlier study, DEEP was shown to reduce state-anxiety, providing a relaxed state that remained around 2 h (Bossenbroek et al., 2020), which was underlined by the expectations of our participants. Participants indicated that several working mechanisms of DEEP that were found to be of possible added value can also be found in other eHealth technologies, such as mindfulness and deep breathing. Consequently, apps might be a cheaper alternative for VR interventions such as DEEP. Research has indeed shown that mindfulness apps that focus on breathing exercises and meditation also show promising results in treating forensic psychiatric patients (Bostock et al., 2019; Walsh et al., 2019). Additionally, the study conducted by Weerdmeester et al. (2021) found regular breathing apps as effective as DEEP for undergraduates, while remaining free and easy to use. Our study showed that while apps seem to be fitting as well, DEEP seems to be more suitable for a forensic setting: both healthcare providers and patients stated that mindfulness apps often focused too much on spirituality and still require reflective and reading skills of its user. Other studies found this as well, stating that many apps require cognitive and digital skills and are based on inwardness, reflection, and introspection (Howells et al., 2010; Kip et al., 2019c; Kip and Bouman, 2020). Even though some breathing apps are less language-based and less focused on spirituality, especially unmotivated forensic psychiatric patients might need more than just an app to feel motivated and engaged to work on their deep breathing. This expectation is strengthened by the study of Weerdmeester et al. (2021), in which it was found that the engagement of DEEP users increased, while no change in engagement was found in users of a breathing app. By using an experience-based and gamified approach, DEEP might offer a unique and engaging way to introduce principles of mindfulness and diaphragmatic breathing to forensic inpatients. However, it is important to further evaluate DEEP to study whether DEEP can be effective for the forensic inpatient care, confirming the expectations of this study. Moreover, future research could explore if mindfulness apps could provide an effective, fitting, and cost-efficient alternative to DEEP in treatment of complex patient populations.

### 4.3. Usage of DEEP: structural vs. ad hoc

Some healthcare providers and patients would like to use DEEP structurally to train breathing skills, for example on fixed days. Others preferred to use DEEP *ad hoc*, on moments where patients felt they could benefit from DEEP. When used structurally, DEEP can be used to teach forensic patients deep breathing skills as a coping-strategy, allowing them to deploy these strategies when they feel anxious or angry. In this way, patients can acquire the deep breathing skills on good days when they have enough mental space to learn, ensuring that on bad moments, they can cope with their negative emotions. Regarding the *ad hoc* approach, DEEP is not used during regular treatment sessions, but can be used on the patients’ initiative when they already feel elevated levels of negative emotions such as anger, stress or anxiety and need a way to escape and relax. This approach might increase the sense of self-management and ownership of patients, as they can decide when they would like to use DEEP and if they would like to use it alone or with their healthcare provider. While forensic psychiatric inpatients are often treated involuntarily, empowering them to take an active role in their care seems important for it to be effective (Senneseth et al., 2022). An increase of ownership and self-management might also motivate and engage patients to use DEEP as an intervention, making it important to adapt it to the needs of patients in helping them reach their treatment goals (O’Leary et al., 2015). In conclusion, DEEP is an intervention that can be used in diverse ways, depending on the needs and preferences of patients. Future research is needed to explore the efficacy and feasibility of both structural and *ad hoc* ways of using DEEP.

### 4.4. Implementing DEEP in forensic mental healthcare

**FIGURE 3:**
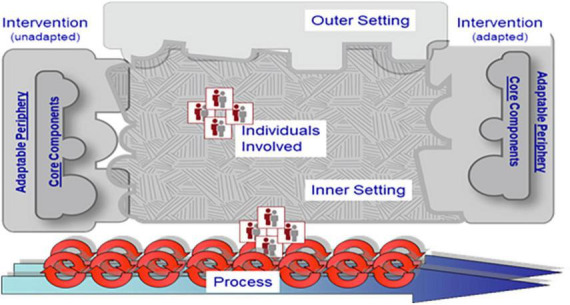
The Consolidated Framework for Implementation Research (CFIR). Reproduced with permission from the CFIR Research Team-Center for Clinical Management Research (https://cfirguide.org).

This study showed the importance of a structural, thorough implementation of DEEP. This is in line with multiple studies that show that the implementation of eHealth is often complex and requires a thorough approach (Schreiweis et al., 2019; Kouijzer et al., 2023). Both healthcare providers and patients provided insight into the factors that are expected to be important for successful implementation of DEEP in forensic inpatient clinics. These factors belong to various levels, ranging from individual patients to the organization. The identified factors are in line with the domains of the *Consolidated Framework of Implementation Research*, which has been used often within healthcare settings (Damschroder et al., 2009, 2022). The CFIR is visually displayed in Figure 3. In this study, multiple advantages and barriers were mentioned about the design and working mechanisms of DEEP regarding the *intervention*, (e.g., the appealing but overwhelming design of DEEP) which might affect its long-term uptake. Our findings are in line with earlier studies that highlight the importance of overcoming a “one-size-fits-all approach” and tailoring the design of an intervention to its end-users (Polaschek, 2011; Wilson et al., 2017). Therefore, it seems to be important to further develop and adjust the hardware and software of DEEP to fit the needs and skills of forensic inpatients and their healthcare providers. This study also suggests the importance of including the *inner setting* and its *individuals* in the implementation process by not only explore the needs of the end-users (e.g., patients), but also those of managers and healthcare providers. This is important because they are the gateway keepers when introducing new technologies to their patients. Other studies point out the importance of involving a range of stakeholders in implementation as well, to account for all relevant perspectives (Marcu et al., 2011). Additionally, the results of this study show the importance of providing practical and content-related support to the healthcare providers and patients during the implementation *process.* This is supported by earlier research that emphasizes the importance holistic implementation approach, with attention to the organizations, patients, and healthcare providers (Kip et al., 2020b). Therefore, before starting the implementation process of DEEP, all needs of the organization, its employees and the patients should be considered to prevent practical barriers, such as the lack of a room for DEEP, to hinder the implementation. According to the CFIR framework, the role of the *outer setting* is essential for implementation, such as beneficial policies and (financial) incentives by government organizations and other external stakeholders to increase the uptake of innovative technologies (Kirk et al., 2016; Ross et al., 2016). The factors related to the outer setting were hardly identified in this study, underlining the need for future research to paint a more comprehensive picture of the wider context surrounding the organization (Christie et al., 2018). Finally, a recent study on the implementation of VR in healthcare highlights the importance of creating a systematic implementation plan, with concrete strategies and objectives linked to clear implementation outcomes (Kouijzer et al., 2023). In our study, participants clearly identified the importance of practical resources, such as room, time and support for caregivers to use DEEP with their patients. In future research, it remains necessary to further study DEEP in forensic psychiatric care to thoroughly and systematically investigate the process of implementing DEEP. Ideally, a comprehensive, multi-level implementation plan with concrete implementation strategies to address possible implementation obstacles has to be created. In conclusion, this study provides a starting point for implementation by identifying expected barriers and facilitators, and thus serves as a first step for future implementation research.

### 4.5. Ethical considerations

When conducting research in forensic psychiatric settings, attention must be paid to the informed consent of forensic psychiatric patients, as most of them receive mandatory treatment (Ligthart et al., 2022a,b; Meynen, 2022). Especially due to the obligatory nature of treatment, it is essential that patients are able to voluntarily provide a fully informed consent. To achieve this, patients who were interested in participating were given an information letter and were verbally informed about DEEP, its mechanisms and the purpose of this study. Specific attention was paid to providing information in a way that was suitable for their cognitive and literacy skills. Furthermore, engaging and immersive VR environments can become triggering when being used in forensic mental healthcare (Kip et al., 2019b). In DEEP, users do emerge themselves in a surrealistic, dreamlike environment. While in this study, no negative impact of such an environment was identified, it might cause triggering effects for some patients. This highlights the importance of continuously paying attention to the experiences of patients that use DEEP, especially to those who are sensitive to light, sounds and psychoses, the inclusion of caregivers and patients in the validation and further development of DEEP.

### 4.6. Strengths and limitations

The main strength of this study is the involvement of both healthcare providers and patients. Both perspectives are important, since both groups are stakeholders of new interventions, while healthcare providers remain often overlooked (Kip et al., 2019a,b). However, DEEP was not available for participants to use in the clinics. This study aimed to overcome this limitation by letting both healthcare providers and patients tryout DEEP before starting with the focus groups and interviews, showing the importance of using concrete products and user-experiences (Beerlage-de Jong et al., 2017; Kip et al., 2022). Even though many healthcare providers and patients stated that they found it useful to try DEEP before participating in the focus groups and interviews, some participants still felt some hesitation to provide definite answers on how and for whom DEEP is of most added value, due to them not being able to use DEEP in their current practice.

In this study, participants volunteered to be included, which can point to selection bias: there is a risk that these participants are already more interested in using new technological interventions, which might not thoroughly represent the forensic inpatient care. We have accounted for this by elaborately focusing on possible disadvantages of DEEP, paying attention to which parts of the intervention might not be suitable for forensic psychiatric inpatients, and focusing on barriers for implementation.

During the interviews it became clear that some patients had trouble with thinking about possible ways DEEP could be of use in their treatment, because of difficulties with abstract reasoning. Therefore, the researcher sometimes decided to shorten the interview by leaving out the more abstract questions about implementation, and by putting more focus on the patient’s first impression of DEEP and on how and when they would prefer to use it, making it more concrete. Adapting the methodology can be seen as good practice in this type of study, to prioritize the wellbeing of the end user (Kip et al., 2022; Schouten et al., 2022). However, this could mean that the perspective of some patients is not fully included. Additionally, due to privacy reasons we did not ask the participants about sociodemographic information such as age, psychiatric diagnoses, or reason for treatment. However, including this information might have resulted in more interesting insights into the way the participants experienced their first impression of DEEP and how they feel DEEP might help them in treatment.

Finally, an earlier version of the game was used in this study, which means that some aspects that may have led to frustration or sub-optimal user experiences have already been improved. Regarding the hardware of DEEP: DEEP is now available on the *Oculus Quest 2* headset and the waist belt and hand controllers are now wireless. However, it is only possible to cast DEEP to a laptop via Wi-Fi connection, making it more complex for healthcare providers to support patients during the game. DEEP is still under development, which means that the hardware and software keeps being expanded and improved. However, this study did underline the importance of keeping its user engaged, while remaining user-friendly and not too overwhelming. This needs to be addressed in future versions of the game to make it suitable for use in a forensic setting.

## 5. Conclusion

With its experience-based and gamified design, DEEP could be of added value for forensic mental healthcare, particularly by providing an appealing and engaging way to teach deep breathing to forensic psychiatric inpatients with low treatment motivation. According to patients and healthcare provider, DEEP can be used both structurally within treatment and *ad hoc* on patients’ initiative. Our study showed that it is important to include patients and healthcare providers in the evaluation and implementation of DEEP from the start. To account for all relevant perspectives and to ensure successful integration in current care pathways, there is a need for a multi-level approach when implementing DEEP. This approach should consider the intervention, the wider forensic context, the ethical considerations and the individuals that are possible end-users of DEEP. These factors can be used to formulate clear implementation strategies, such as a practical DEEP-training and clear protocols for caregivers on when, how and for whom DEEP is to be used, as well as to further evaluate DEEP in forensic mental healthcare. If implemented well, DEEP can offer new, experience-based ways to provide forensic psychiatric patients with strategies to better cope with their negative emotions, such as stress and anger and thus prevent recidivism.

## Data availability statement

The raw data supporting the conclusions of this article will be made available by the authors, without undue reservation.

## Ethics statement

The studies involving human participants were reviewed and approved by the Ethics Committee of the University of Twente (Behavioral, Management and Social Sciences, nr. 21119). The patients/participants provided their written informed consent to participate in this study.

## Author contributions

HK and LK contributed to the design and planning of the study and analyzed the focus group and interview data. LK collected the focus group and interview data. All authors contributed to the interpretation and reporting of the results and approved the manuscript.

## Appendix

**Appendix A:** Semi-structured topic list for focus groups with healthcare providers.

To what extent is DEEP useful for your patient population?	RQ1
What kind of benefits could it have?	RQ1
What do you see as the potential added value of DEEP?	RQ1
Why do you expect DEEP to work?	RQ1
What downsides or difficult issues do you see? Any barriers?	RQ1
For what types of patients would you want to use this, and when?	RQ2
Are there also patients for whom you would not want to use DEEP?	RQ2
If we want to use DEEP in the clinic, what should we take into account?	RQ2-4
○ Are there certain things related to patient characteristics? ○ What do you need? How would you like to be supported? ○ What should the organization do? ○ Are there certain things in DEEP itself that need to be clearer/better? ○ Do you have any suggestions for improvement?	
What else would you like to know about DEEP? What are you curious about?	
Are there any other questions, comments, or ideas that are important for us to consider?	

**Appendix B:** Semi-structured interview scheme for interviews with patients.

Would you want to use DEEP yourself? Why or why not?	RQ1
What benefits do you think DEEP could have for you?	RQ1
How could it help you? And for other patients admitted here?	RQ1-2
What possible downsides of DEEP do you see? What would be difficult, hard or not fun?	RQ1
When would you prefer to use DEEP? At what moments or periods?	RQ3
And when would you really not want to use DEEP?	RQ3
Imagine that we would use DEEP at your clinic. What should we take into account?	RQ4
○ What kind of information would you like to have before using DEEP? ○ How would you like to be helped when using DEEP? ○ What do you think is important for your care providers? How would you like to involve them? ○ What do you expect from us as researchers? ○ If you look at DEEP now, are there certain things you would like to see differently or better? Do you have any tips to improve it further?	
Do you have any further comments, questions or ideas?	

**Appendix C:** Included healthcare providers and patients per forensic organization (*N* = 24; *N* = 13).

	Healthcare providers	Patients
Transfore	14	4
FPK	3	2
FPA	3	2
Forence	8	0
**De Woenselse Poort**	**10**	**9**
Balans	9	1
Keer	0	2*
Verbinding	1	6
**Total**	**24**	**13**

*At this clinic no focus groups were organized. However, during the introduction of DEEP and interviews with patients of other clinics two patients who received treatment at Keer saw the VR-set and spontaneously asked to be included themselves.
